# Zeolite/LDH Composites as Additives for Light Olefin Production by Catalytic Cracking of Heavy Oil Fractions

**DOI:** 10.1021/acsaem.5c02910

**Published:** 2025-11-14

**Authors:** Chadatip Rodaum, Chularat Wattanakit, Avelino Corma, Cristina Martínez

**Affiliations:** † Department of Chemical and Biomolecular Engineering, School of Energy Science and Engineering, 423058 Vidyasirimedhi Institute of Science and Technology, Rayong 21210, Thailand; ‡ 16774 Instituto de Tecnología Química (UPV-CSIC), Universitat Politècnica de València-Consejo Superior de Investigaciones Científicas, Avda. de los Naranjos s/n, Valencia 46022, Spain

**Keywords:** zeolite/LDH, FCC additive, catalytic cracking, heavy oils, propylene

## Abstract

Among the possible strategies for the enhancement of light olefin production in the catalytic cracking of heavy oils, the use of functional additives has proven to be an effective approach. Herein, we present a composite material (Z5-LDH) derived from medium-pore zeolite ZSM-5 (Z5) and layered double hydroxide (LDH)-based mixed oxides as a potential additive for catalytic cracking of heavy oils. This material shows enhanced selectivity to light olefins within the gaseous C3–C4 fraction. The Z5-LDH composite features a core–shell structure, with the zeolite forming the core, which is surrounded by an LDH shell. It exhibits reduced Brønsted acidity compared to pure ZSM-5 and surface basicity provided by the basic oxides formed upon calcination of the LDH. The catalytic performance of the composite has been compared with that of pure ZSM-5 for catalytic cracking of vacuum gasoil (VGO). When used as an additive, the composite yields a significantly higher olefin content and selectivity within the C3–C4 gas fraction than a commercial USY base catalyst alone (17.2% compared with 11.3 wt %, respectively). Furthermore, the propylene/propane and butenes/butanes ratios obtained with the Z5-LDH additive are substantially higher than those obtained with the pure ZSM-5 additive. The enhanced selectivity to C3–C4 olefins is related, on the one hand, to the lower density and acid strength of the Brønsted acid sites in Z5-LDH relative to ZSM-5, which minimizes secondary reactions such as hydrogen transfer. On the other hand, the basic mixed-metal oxide shell, derived from the LDH, inhibits the readsorption of the C3–C4 olefins produced, limiting their further conversion and promoting light olefin selectivity. The advantages of the Z5-LDH composite as a fluid catalytic cracking (FCC) additive have been demonstrated not only for conversion of conventional VGO but also in the cracking of heavier feeds, such as atmospheric residue.

## Graphical abstract

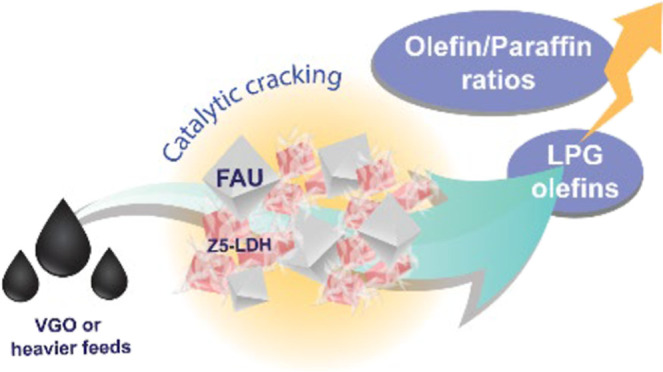

## Introduction

Light olefins, including ethylene, propylene, and butenes, are key building blocks in the petrochemical industry and are used for manufacturing plastics and chemicals.^1,2^ Although the development of on-purpose olefin production routes, such as direct propane dehydrogenation, ethene-to-propene, methanol-to-olefins, olefin metathesis, or catalytic cracking of low-value butene fractions, is receiving increasing attention in the last decades,^3–5^ currently, light olefins are still mainly produced through steam cracking of hydrocarbons or via fluid catalytic cracking (FCC) of heavy oil fractions such as vacuum gas oil (VGO), crude oil, or residues.^6–9^ Steam cracking is an energy-intensive process, often leading to the formation of large amounts of coke and poor selectivity toward the desired light olefins, especially when moving from naphtha to lighter feeds such as natural gas. On the other hand, FCC is a highly flexible process, capable of handling different heavy oil fractions and selectively adjusting product distributions, and the second-largest supplier of light olefins, primarily propylene, after steam cracking.^8,10,11^

The FCC catalyst is formed by different components among which the main active component is an ultrastable form of zeolite Y (USY) with a large pore FAU structure.^9^ Traditional USY zeolites present secondary meso- and macroporosity obtained by the combination of acid and hydrothermal postsynthesis treatments.^9^ However, other strategies for the preparation of hierarchical Y zeolite-based catalysts, with increased accessibility and presenting increased activity due to the presence of additional mesoporosity and improved acidic properties, have also been described.^12^ Regarding the FCC process, several factors influence the yield to light olefins, such as the properties of the main catalyst, the use of additives, process conditions (reaction temperature and residence time), and feedstock properties.

To date, various strategies are being developed from both the catalyst and the process point of view, in order to enhance the production of light olefins.^13,14^ One of the most cost-effective and efficient routes to enhance the yield of these products is the addition of catalyst additives to the base FCC catalyst.^9^ The presence of these additives, usually based on medium pore ZSM-5 zeolite [MFI], contributes to the process by selectively cracking gasoline fraction hydrocarbons, increasing in this way the yield to propylene and butenes.^15–18^

Besides ZSM-5, other zeolites have been described in the literature as potential FCC catalyst additives, such as IM-5, SSZ-74, ferrierite, MCM-22, ITQ-13, and ITQ-7.^19–23^ Still, the medium pore ZSM-5 zeolite is the one used in commercial FCC propylene boosting additives due to its high efficiency and affordable production costs.^8,24,25^

Although ZSM-5 additives can selectively crack the gasoline hydrocarbons into lower olefins, the produced alkenes are unstable and can be easily consumed via undesired consecutive reactions such as hydrogen transfer, oligomerization, and cyclization reactions, leading to a decrease in their yield.^17,26^ Thus, there is still room for improving the zeolite additives in order to suppress these undesired secondary reactions and to increase the light olefins production in the FCC unit. One of the strategies followed to improve the catalytic performance of ZSM-5-based additives is to incorporate some components such as phosphorus, alkali or alkali earth metals, and rare earth metals, aiming to reduce the number of acid sites and/or to modify their acid strength distribution.^6,27,28^ Conventionally, the incorporation of these metals is done by impregnation, in situ incorporation, or ion-exchange methods.^29–31^ However, these procedures present some drawbacks such as metal aggregation, resulting in pore blocking or limited metal contents. A highly interesting alternative approach to achieve high metal dispersion on zeolite surfaces is by synthesizing zeolite/LDH composite materials.^32,33^

Layered double hydroxides (LDH), also known as anionic clays or hydrotalcite-like compounds, are synthetic materials with a layered crystalline structure. Their structure is composed of positively charged metal hydroxide layers intercalated with negatively charged interlayer anions. The general formula for LDH can be expressed as [M^2+^_1–*x*_M^3+^_*x*_(OH)_2_]­[A^*n*–^]_*x*/*n*_·*w*H_2_O, where M^2+^ and M^3+^ represent divalent and trivalent metal cations, respectively, A^*n*–^ represent exchangeable anions, and w refers to the water molecules.^34,35^ Due to the unique properties of these materials, such as their anion-exchange capacity, high surface area, and tunable acid–base properties, a wide range of different applications have been described, e.g., drug delivery, gas storage, and catalysis.^36–42^

The development of zeolite/LDH composite materials was first proposed by Chen et al.^43^ and has been further explored by our group. Recently, we have shown that these hybrid materials can be used as catalysts to enhance light olefin production in the catalytic cracking reaction of *n*-pentane and in the dehydration of bioethanol to ethylene.^33,44^ Because of their core–shell structure, with the zeolite core surrounded by the mixed oxide shell, and their tunable acid–base properties, it is possible to reduce the number of acid sites of the zeolite’s external surface while generating basic sites on the outer shell that suppress the readsorption of reactive olefins. Basic surfaces, such as the mixed MgAl oxides formed by calcination of the LDH, are electron-donating and therefore have less affinity for electron-rich olefins. Lower adsorption enthalpy due to weak pi-interactions results in lower equilibrium affinity, lower surface coverages, and reduced readsorption probability, leading to a decreased extension of undesired reactions and formation of byproducts. The increased olefin selectivity when using basic catalysts/supports has been described not only for catalytic cracking of hydrocarbons,^27,33,45^ or ethanol dehydration,^44^ but also in other processes such as olefin metathesis,^46^ catalytic pyrolysis of crude oils,^47^ steam cracking,^48^ or 1-butene cracking to propene.^49^

Here, we present the catalytic performance of a ZSM-5/LDH (Z5-LDH) composite as a FCC catalyst additive. The composite, which combines the ZSM-5 zeolite in the core covered by the LDH-derived mixed oxides, is highly selective to olefins within the C_3_–C_4_ fraction and yields less propane and butanes as compared to a pure ZSM-5 additive. The benefits of the composite in terms of light olefins production are demonstrated, not only for catalytic cracking of a conventional VGO but also for conversion of a heavier atmospheric residue.

## Experimental Section

### Catalyst Preparation

The Z5-LDH composite was synthesized following a procedure adapted from previous work.^33,44^ The ZSM-5 zeolite used is commercial (CBV5524G, Zeolyst Int.), supplied in its ammonic form, and denoted as Z5-NH_4_. More details on the synthesis procedure of the composite and the pure LDH can be found in the Supporting Information.

To be used as an acid catalyst, the as-prepared composite (Z5-LDH-as) was converted into its final protonic form by means of an ion-exchange process with 0.1 M NH_4_NO_3_ at 80 °C for 2 h under vigorous stirring, followed by filtration, washing, and drying. The procedure was repeated three times. Finally, the obtained sample was calcined at 550 °C for 2 h at a heating rate of 2 °C·min^–1^. The final catalyst was designated as Z5-LDH.

Details on the preparation of Na^+^-exchanged ZSM-5 (Na-Z5) and on the hydrothermal treatment applied to the composite are given in the Supporting Information.

### Catalyst Characterization Techniques

The structure and crystallinity of the zeolites were determined by using powder X-ray diffraction (PXRD), and the Si/Al ratio was determined by inductively coupled plasma (ICP). The morphology of the samples and elemental composition mapping was studied by field emission scanning electron microscopy (FE-SEM) and energy-dispersive X-ray spectroscopy (EDS). Textural properties were obtained from the nitrogen adsorption isotherms measured at 77 K. The acid properties of the samples were evaluated by FTIR spectrometry using pyridine as the probe molecule and by NH_3_-TPD. Details of the characterization techniques are given in the Supporting Information.

### Catalytic Performance

The catalytic cracking experiments were performed in a microactivity test (MAT) unit at 520 °C and 30 s time on stream (TOS). When used as additives for the catalytic cracking of the heavy oil fractions, a commercial USY zeolite, denoted as FAU, was used as the main catalyst, and Z5 and Z5-LDH were added in a 20 wt %. For studying the intrinsic contribution of the ZSM-5 and LDH-derived mixed oxides to VGO cracking, in the absence of the FAU base catalysts, four different cases were compared: the calcined ZSM-5 zeolite alone, Z5, the final acid Z5-LDH composite, a physical mixture of Z5 and calcined LDH, Phy, and a double-bed configuration (2-Bed) with the calcined LDH in the first bed (top) and Z5 in the second one (bottom). A detailed description of the catalytic tests and the product analysis is provided in the Supporting Information.

In this work, two different feedstocks were used: a VGO and an atmospheric residue (ATM residue). The properties of the feeds are enclosed in Table S1.

## Results and Discussion

### Characterization of Catalyst Additives

The ZSM-5 zeolite/LDH composite material (Z5-LDH) composites presented here were prepared following the procedure previously described by Rodaum et al. in refs 33 and 44, a methodology that enabled the successful synthesis of the material with a core–shell structure, according to the electron microscopy measurements and EDS elemental mapping and to the XPS depth profile analysis in Si 2p3/2 and Mg 2p3/2.^33^ In good agreement with these previous results,^33,44^ the powder XRD pattern of the as-synthesized composite (Z5-LDH-as, Figure 1a) exhibits well-defined characteristic peaks of both ZSM-5 (2θ = 7.9°, 8.8°, 23.1°, 23.9°, and 29.8°) and LDH (2θ = 11.4°, 34.7°, 39.0°, and 46.4°).^33,39,50,51^ After the ion-exchange and calcination steps, the peaks corresponding to the parent LDH-as material are no longer present in the diffractogram corresponding to the final material (Z5-LDH, Figure 1b). The decomposition of the intralayer hydroxyl and carbonate anions in the LDH-as structures during the high temperature treatment leads to the formation of the mixed Al_2_O_3_–MgO oxide, as evidenced by the appearance of two broad diffraction peaks at 2θ = 35° and 43°, observed in both the calcined LDH and the calcined composite (see Figure 1b).

**Figure 1:**
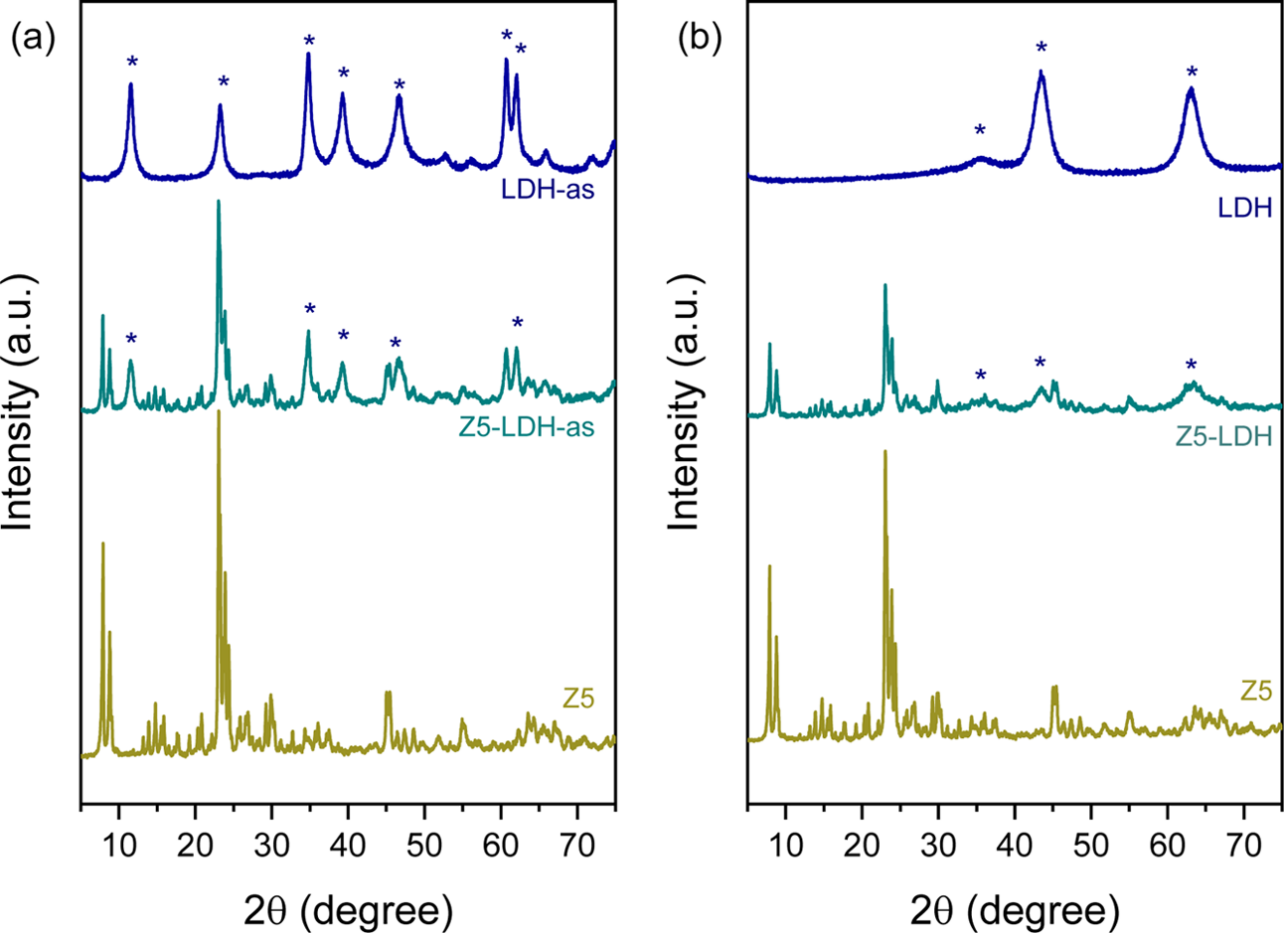
Powder XRD patterns of the catalysts before (a) and after (b) the ion-exchange and calcination processes.

The estimated LDH content is around 38% (see Table S2) based on the ICP results. This is in good agreement with the relative crystallinity of Z5-LDH (61.4%) as compared to that of pure commercial ZSM-5 (Z5) due to the incorporation of the mixed metal oxide to the zeolite crystals (Table S3). Thus, according to these results, the Z5-LDH composite is formed by ∼62% zeolite and ∼38% LDH.

SEM was applied to study the morphology of the synthesized samples. The images revealed that pure commercial ZSM-5 (Z5) is formed by agglomerations of nanocrystals (Figure 2a), whereas pure LDH exhibits typical flower-like structures (Figure 2b). Regarding the synthesized Z5-LDH composite, only flowerlike structures are observed, suggesting that the zeolite crystals are fully covered by the LDH material (Figure 2c,d).

**Figure 2:**
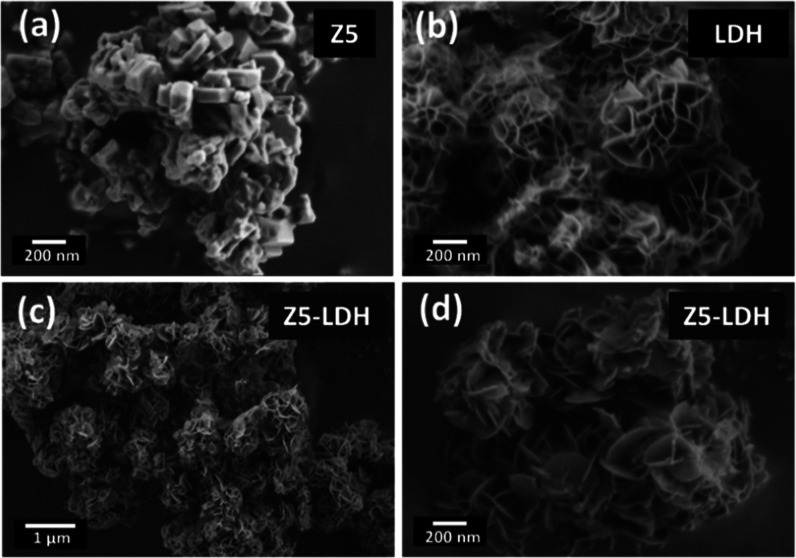
FE-SEM images of pure ZSM-5, Z5 (a), pure LDH (b), and Z5-LDH composite (c,d).

Moreover, the elemental composition mapping (Figure 3) illustrates a homogeneous overlapping distribution of Si (green) and Mg (red), representative of the ZSM-5 and LDH materials, respectively, confirming the successful synthesis of Z5-LDH with uniformly grown LDH on the surface of the zeolite crystallites.

**Figure 3:**
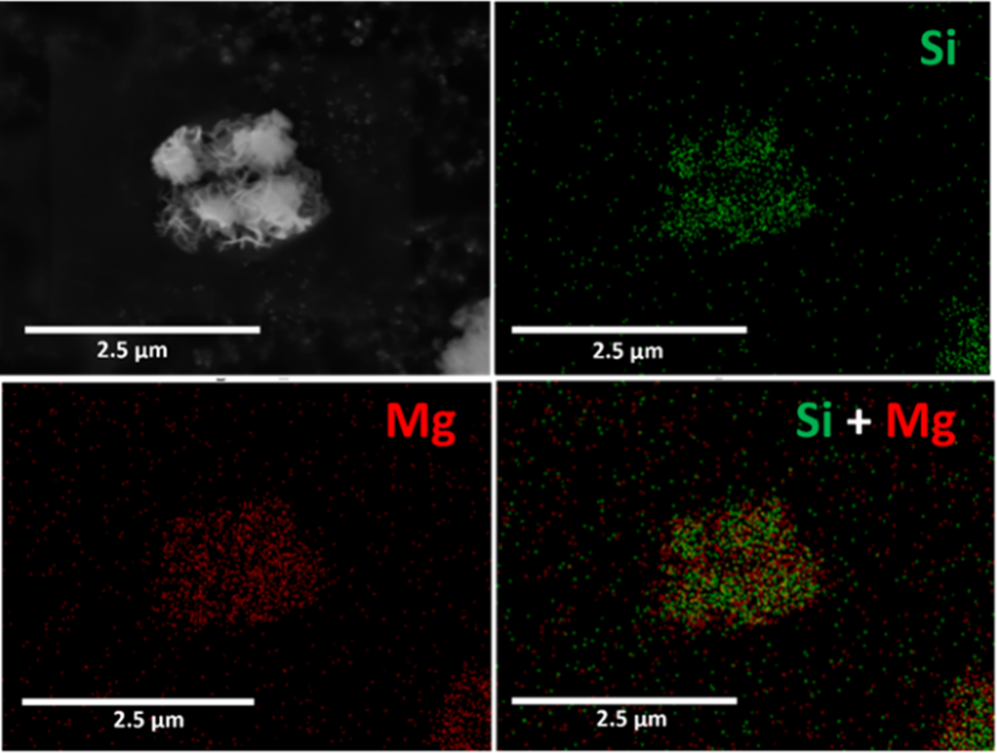
EDS elemental mapping images of Z5-LDH.

Regarding the textural properties of the samples, the type I isotherm and high N_2_ uptake at low relative pressure observed for Z5 are typical of a microporous zeolite, whereas the calcined LDH sample presents a type IV isotherm, indicating the presence of mesopores (see Figure S1). The Z5-LDH sample exhibits a combination of both type I and type IV isotherms with a hysteresis loop, indicating the contribution of both micro- and mesoporosity, as could be expected.

This results in a lower BET surface area (*S*_BET_) and micropore volume (*V*_micro_) of the Z5-LDH composite as compared to that of the pure ZSM-5 zeolite (Table S4) and in a higher mesopore volume, which can be due not only to the presence of the LDH-derived material on the ZSM-5 surface but also to the partial desilication of the zeolite during the first step of the preparation procedure.^33,44^

The combination of the zeolite with LDH-based mixed oxides also affects the acidity of the samples. As described previously for this type of composites,^33,44^ the total amount of acid sites decreases as compared to that of the pure ZSM-5 according to NH_3_-TPD measurements (see Figure S2), and the type of acid sites is also different, as observed by FTIR spectroscopy combined with the adsorption of pyridine. In fact, although all spectra present the two characteristic bands at 1545 and 1450 cm^–1^, assigned to the probe molecule chemisorbed on Brønsted acid sites (BAS) and Lewis acid sites (LAS), respectively,^52,53^ the relative amount of BAS and LAS differs significantly when comparing the composite and the parent ZSM-5. As illustrated in Figure S3, the commercial ZSM-5 (Z5) presents a higher BAS density and only a small amount of LAS, whereas the spectrum corresponding to the Z5-LDH composite exhibits a significantly larger signal at 1450 cm^–1^ and a decreased band at 1545 cm^–1^ in comparison to the pure zeolite, indicating that the presence of the LDH increases the Lewis acidity of the composite and reduces its Brønsted acidity (see Table 1). Moreover, not only the total amount of BAS decreases but also the proportion of the stronger sites, which are able to retain pyridine at increasing temperatures (see Table 1).

**Table 1:** Acidity of the Commercial ZSM-5 (Z5) and Z5-LDH as Determined by FT-IR Combined with Pyridine Adsorption–Desorption^a^

	Lewis acid sites (LAS)^a^ (μmol/g of zeolite)			Brønsted acid sites (BAS)^a^ (μmol/g of zeolite)		
sample	*T* = 150 °C	*T* = 250 °C	*T* = 350 °C	*T* = 150 °C	*T* = 250 °C	*T* = 350 °C
Z5	44	26	16	345	323	259
Z5-LDH	506	254	146	290	228	150
Phy	227	100	83	327	259	221

a μmol of pyridine retained after desorption at increasing temperatures.

The increase in the total number of LAS can be directly related to the Lewis acidity of the mixed oxides forming the shell of the composite.^54,55^ The loss in Bronsted acid site density can be due to partial dealumination of the zeolite during the preparation of the final acid Z5-LDH material (treatment under basic conditions, ion exchanges, and calcination), dealumination that would generate additional Lewis acidity associated with the extra-framework Al species. In order to better understand the changes observed, a physical mixture of ZSM-5 and LDH in a 62:38 wt/wt proportion (Phy in Table 1) has been prepared, and the nature and number of acid sites of the composite have been compared with those of the composite. It can be seen that the total amount of Bronsted acid sites measured on the physical mixture is higher than that of the Z5-LDH material and closer to that of the pure Z5 when the number of sites is referred to the amount of zeolite (see Table 1). Regarding the number of LAS, it is significantly higher for the composite suggesting a large dispersion of the basic solid on the external surface of the zeolite’s crystals.

The presence of the LDH coverage on the external surface of the ZSM-5 zeolite also provides a basic character to the composite, as mentioned in the Introduction section. Indeed, the basicity of these materials, measured by CO2-TPD, is confirmed by the appearance of three peaks in the temperature-programmed desorption profile, a first one in the range of 100–300 °C attributed to weak basic sites, a second peak in the range of 350–550 °C assigned to medium and strong basic sites, and a peak at higher temperature (>570 °C) indicating the presence of very strong basicity.^33^

It is reasonable to expect that the changes in the acid–base properties of the Z5-LDH composite may play an important role in the yields and selectivity to light olefins obtained by the catalytic cracking of heavy petroleum oils.

### Catalytic Cracking of Vacuum Gas Oil (VGO) over the Different Additives

Before testing Z5-LDH as an additive for heavy oil cracking, the intrinsic contribution of the mixed oxides present in the composite to the catalytic performance was studied. This was approached by comparing the activity of the additives alone, tested in the absence of the USY base catalyst. Thus, the calcined ZSM-5 zeolite alone, Z5, and the final acid Z5-LDH composite were studied as the main catalysts in the catalytic cracking of a VGO whose properties are given in Table S1. For comparison purposes, a physical mixture of Z5 and calcined LDH (Phy) and a double-bed configuration (2-Bed) with the calcined LDH in the first bed (top) and Z5 in the second one (bottom) were tested under the same conditions. For this comparison, all tests have been performed at a constant zeolite content of 0.3 g (see additional details in the Supporting Information). As shown in Figure S4a, increasing the catalyst to oil ratio (C/O) results, in all cases, in an increase in total conversion, and the highest activity is obtained with the 2-bed configuration, probably because some precracking of the heavy VGO molecules occurs on the mixed oxides located in the upper bed, favoring further conversion of the primary products on the medium pore Z5 zeolite downstream. The lowest conversion is obtained with the Z5-LDH composite, probably because of its reduced acidity. Under these conditions, it is also less selective to gasoline and most selective to diesel (see Figure S4b–e).

A closer analysis of the light olefin selectivity is done by comparing the yields obtained at similar conversion levels of about 33–35% (Figure 4). Although the selectivity to gases is comparable for all additives (see Figure S4b), the Z5-LDH composite presents the highest yield to total olefins (14.5% as compared to 9.1% for the pure Z5, 9.3% for the double bed, and 10.8 for the physical mixture), especially to propylene and iso-butene. This results in significantly higher propylene/propane (C_3_^=^/C_3_) and butene/butane (C_4_^=^/C_4_) ratios for the composite (Figure 5).

**Figure 4:**
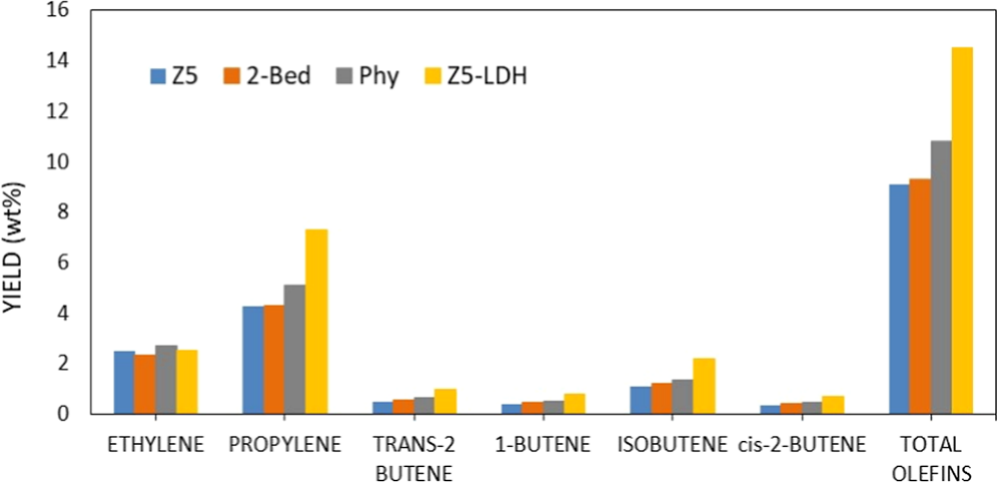
Comparison of light olefin yields between different catalysts at a VGO conversion of 33–35% in VGO cracking reaction at 520 °C and 30 s TOS.

**Figure 5:**
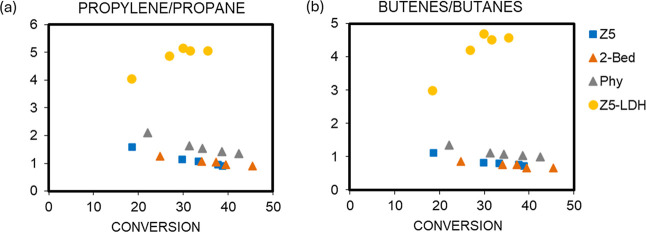
Propylene/propane (a) and butenes/butane (b) ratios obtained with different catalysts in VGO cracking reaction at 520 °C and 30 s TOS.

It is well known that the reduction of the number of BAS decreases the extension of bimolecular hydrogen transfer reactions, improving, therefore, the selectivity to light olefins within the gases. Thus, the increase in the light olefin yield of Z5-LDH as compared to the pure Z5 zeolite could be attributed to its lower BAS density and reduced acid strength. In order to confirm if these are the only factors influencing the light olefin yield, an additional experiment was performed by using a Na^+^-exchanged ZSM-5 (Na-Z5), with a number of BAS comparable to that of the Z5-LDH composite (see Table S5). Activity and overall selectivity to gases, liquid fuels, and coke obtained with the two catalysts are similar, indicating comparable overall cracking performance due to a similar number of active sites (see Figure S5a). However, the composite outperformed Na-Z5 in terms of light olefin selectivity, especially when comparing propylene and isobutene (see Figure S5b). The reduced formation of propane and isobutane is clear evidence of a reduced hydrogen transfer capacity in the case of the Z5-LDH catalyst, as a result of the presence of the basic mixed oxides on the surface of the ZSM-5 crystals. At a similar conversion level (∼30%), both Na-Z5 and Z5-LDH catalysts show the same trends in the overall product distribution, including diesel, gasoline, gases, and coke as shown in Figure S5a. However, comparing the product selectivity within the gas phase, it is evident that the Na-Z5 catalyst provides lower propylene and butene selectivity than Z5-LDH (Figure S5b) and lower C_3_^=^/C_3_ and C_4_^=^/C_4_ ratios (Figure S6a,b), despite having equivalent BAS density. The presence of the LDH component can also be discarded as the only component responsible for the higher olefin selectivity obtained with the composite. In fact, the increase in the propylene and butenes yield when the calcined LDH is added to the zeolite, either in a separated bed or in a physical mixture, is significantly lower as compared to that obtained with the Z5-LDH composite (Figure 4).

These results suggest that there might be an additional factor affecting the olefin selectivity obtained with the Z5-LDH composite, besides the mere reduction of BAS or the individual contribution of the mixed oxides. This enhancement can be attributed to the specific disposition of the basic metal oxides derived from LDH covering the zeolite. Once the olefins are formed within the Z5 crystals and egressed from the Z5-LDH composite into the reaction media, the surface basicity of the LDH shell will prevent their readsorption and consumption in undesired side reactions.^33,44^ Based on these findings, it can be expected that the use of Z5-LDH as an additive in heavy oil cracking will enhance the production of light olefins.

### Catalytic Cracking of Heavy Petroleum Oils over Different Additive Containing Catalysts

The preliminary results obtained in the previous section, where the Z5-LDH composite has been compared to pure ZSM-5 as the main catalyst in the cracking of VGO, have clearly demonstrated the benefits of this combination of the acid zeolite and the basic LDH derived mixed oxides in terms of light olefin selectivity. In this section, the two materials will be used in combination with a conventional USY base catalyst, and their effectivity as propylene boosting additives in VGO cracking will be compared. The USY zeolite, supplied by Zeolyst Int., has a unit cell size of 24.24 Å and a molar Si/Al ratio of 30 according to the supplier, and its main textural properties are presented in Table S4. Thus, the catalysts studied in this section are three, a commercial USY zeolite alone (FAU), with no additive, and a combination of FAU with Z5 and Z5-LDH (FAU/Z5 and FAU/Z5-LDH, respectively) where the additive (20 wt %) is mixed with USY as independent particles in a single bed. The performance of FAU, FAU/Z5, and FAU/Z5-LDH as catalysts for cracking of a VGO is compared in Figure S7. In all cases, the conversion increased in the range of 63–94% when increasing C/O ratio from 0.15 to 0.67, with only a small contribution of the additives to the conversion degree in the low C/O ranges (Figure S7a).

These results could be expected because the C/O ratios are referred to the amount of the base catalyst, FAU (see the Experimental Section). However, significant differences are observed in the overall product distribution, where the presence of the additives leads to lower gasoline and higher gas selectivities (see Figure S7b–e). The influence of the additive on the product distribution is larger for pure ZSM-5 than for the Z5-LDH composite, and this can also be concluded from the results presented in Figure 6, where the product yields are compared at a similar conversion level. In fact, when comparing conditions of iso-conversion, it is clear that the presence of the additives enhances recracking of gasoline-range hydrocarbons into lighter products such as dry gas (C1–C2) and LPG (C3–C4).

**Figure 6:**
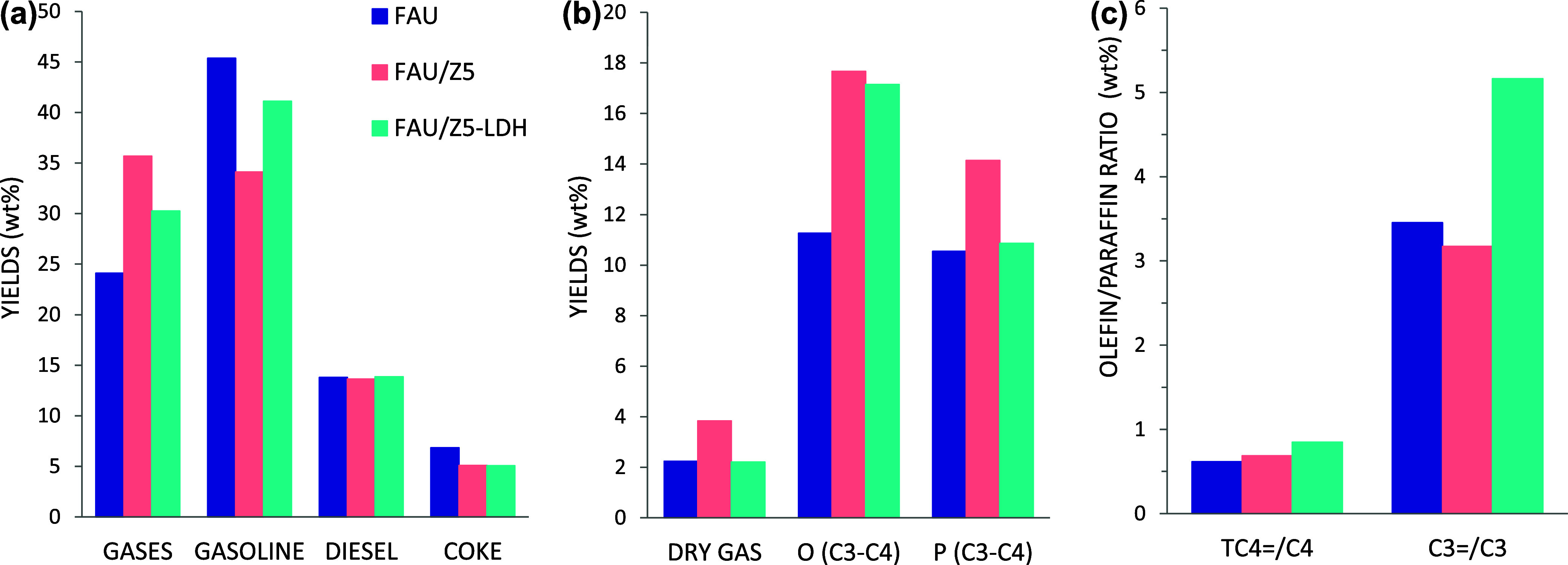
Catalytic performance of the different additives at similar conversion of ∼90%: overall product distribution (wt %) (a), product distribution (dry gas, C3–C4 olefins, and C3–C4 paraffins) in gas fraction (wt %) (b), and olefin/paraffin ratios (c) in VGO cracking reaction at 520 °C and 30 s TOS.

In terms of the product distribution within the gas fraction (Figure 6b), the increased dry gas (C_1_–C_2_) yield obtained with FAU/Z5 as compared to FAU/Z5-LDH is due mainly to the increase in the ethylene yield (see Figure S8a). This could be explained by a larger extent of recracking reactions due to the higher Brønsted acid density in the Z5 additive as compared to Z5-LDH. In the case of the C_3_–C_4_ products, the olefin yields (propylene and butene) obtained with both FAU/Z5 and FAU/Z5-LDH are comparable (17.7 and 17.2 wt %, respectively, see Figure S8a). However, the use of the Z5-LDH composite results in a significant decrease in the C_3_–C_4_ paraffins production (propane and butane) as compared to the pure ZSM-5 (Z5) additive and, consequently, in an important increase of the propylene/propane and the butene/butane ratios (Figure 6c). These differences relate to the lower amount of BAS, which will minimize the hydrogen transfer reactions that saturate the olefins to paraffins, but also, as described in the previous section, to the generation of surface basicity in the Z5-LDH composite. The basic mixed oxides present in the composite shell reduce the readsorption of the light olefins produced, preventing their consumption in undesired consecutive reactions and leading, therefore, to an improved light olefins selectivity.^33,44^

Hydrothermal stability of FCC catalysts and additives is a crucial factor. Thus, the Z5-LDH additive has been treated for 5 h at 750 °C under a 100% steam atmosphere (Z5-LDH-ST), and its catalytic behavior, when added to the commercial FAU catalyst, has been compared with that of the calcined Z5-LDH (see Figure S9). The differences in total conversion and overall selectivity are negligible, confirming the good hydrothermal stability of the composite. Moreover, when repeating the first experiment (C/O = 0.15) after the full series of five experiments, the activity and selectivity are also reproduced.

The promising results obtained encouraged us to further verify the potential advantage of the Z5-LDH composite when applied to the conversion of heavier feedstocks. Thus, the Z5-LDH composite was also tested as a propylene boosting additive in catalytic cracking of an atmospheric (ATM) residue containing a larger bottoms fraction (boiling point >482 °C) and higher concentration of asphaltenes and metals than the previous VGO (see Table S5).

As shown in Figure 7, the product distribution of all samples at an ATM residue conversion of 90% exhibits trends similar to those observed for the VGO cracking reaction described previously. The Z5-LDH additive provides higher yields to C_3_–C_4_ olefins and produces lower amounts of C_3_–C_4_ paraffins as compared to the pure Z5 when converting this heavier feed (see Figure S8b), confirming the high selectivity of the composite-based additives to light olefins, not only when converting conventional VGO but also for the catalytic cracking of heavier residue fractions. Besides increasing the olefinicity within the C_3_–C_4_ fraction, the Z5-LDH-based additive decreases the selectivity of dry gas and coke, two additional advantages as compared to pure ZSM-5.

**Figure 7:**
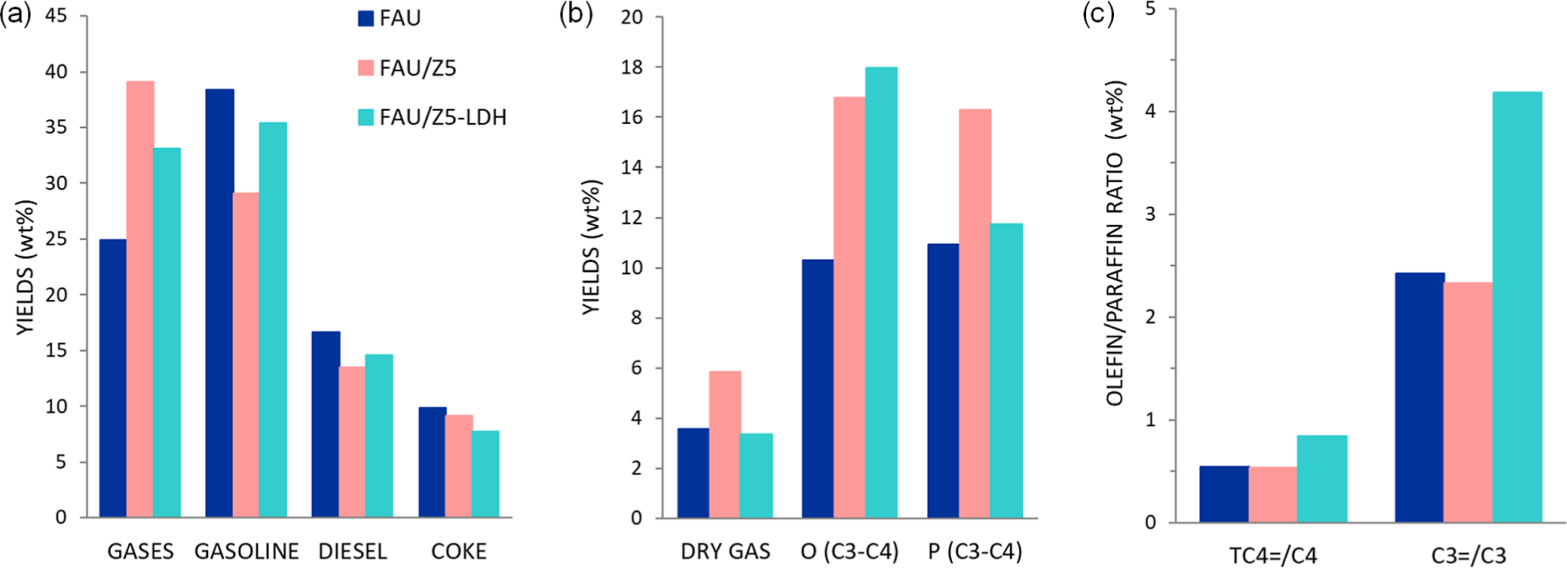
Catalytic performance of the different additives at similar conversion of ∼90%: overall product distribution (wt %) (a), product distribution (dry gas, C3–C4 olefins and C3–C4 paraffins) in gas fraction (wt %) (b), and olefin/paraffin ratios (c) in ATM residue cracking reaction at 520 °C and 30 s TOS.

## Conclusions

A Z5-LDH composite was successfully synthesized following a simple and reproducible procedure. The catalytic performance of this material, after calcination for obtaining the LDH-derived mixed oxides, was compared to that of other ZSM-5-based additives in the catalytic cracking of heavy oil fractions, tested alone or as additives of a conventional USY base catalyst, FAU, at 20 wt % relative to the main catalyst. When tested in the absence of USY, the Z5-LDH composite showed promising results as compared to pure ZSM-5, to a physical mixture, and to a double-bed catalyst of ZSM-5 and calcined LDH, significantly enhancing the production of light olefins, specifically propylene and butene, and reducing the formation of the corresponding paraffins. These positive results led us to test the use of the Z5-LDH composite as an additive in the catalytic cracking process of heavy oils such as VGO and ATM residues, with the aim of increasing the light olefin production. Indeed, the composite shows an increased C_3_–C_4_ olefin selectivity as compared to the pure ZSM-5 (31.5 wt % vs 24.4 wt % in the case of propylene and 25.23 wt % vs 21.09 wt % in the case of butenes) and significantly higher olefin/paraffin ratios, in particular the propylene/propane ratio (5.2 vs 3.2). This behavior can be directly related to the decrease in Brønsted acid site density and acid strength as well as to the specific structure of the composite, with the presence of basic metal oxides in the outer shell of the Z5-LDH material. These factors prevent the readsorption of the primary olefins formed and, therefore, the occurrence of undesirable secondary reactions such as hydrogen transfer, oligomerization, and cyclization, where these light olefins will be consumed. As a result, the use of a Z5-LDH composite as an FCC additive greatly improves the selectivity to C_3_–C_4_ olefins at yields comparable to those obtained with a pure ZSM-5.

## Supplementary Material



## Notes

Detailed description of catalyst preparation, characterization and catalytic testing procedures, characterization of materials (N_2_ adsorption–desorption isotherms, NH_3_-TPD profiles, FT-IR spectra), catalytic results (comparison of different catalyst bed configurations, evaluation of Na-ZSM-5, and evaluation of steamed additives), and characteristics of the industrial feeds employed (PDF)
