# Supplementary material for: Zeolite/LDH Composites as Additives for Light Olefin Production by Catalytic Cracking of Heavy Oil Fractions
Source: ACS Appl Energy Mater. 2025 Nov 14;8(23):17442–50. doi: 10.1021/acsaem.5c02910 (PMC12691186; doi:10.1021/acsaem.5c02910)
Supplement: Supplementary file 1 [file ae5c02910_si_001.pdf]

Supporting Information

Zeolite/LDH composites as additives for light olefin production by catalytic cracking of heavy oil fractions

Chadatip Rodaum [a], Chularat Wattanakit [a], Avelino Corma [b, *], Cristina Martínez [b, *]

[a] *Department of Chemical and Biomolecular Engineering, School of Energy Science and Engineering, Vidyasirimedhi Institute of Science and Technology, Rayong 21210, Thailand.*

[b] *Instituto de Tecnología Química (UPV-CSIC), Universitat Politècnica de València-Consejo Superior de Investigaciones Científicas, Avda. de los Naranjos s/n, Valencia 46022, Spain.*

**Corresponding author. E-mail addresses: cmsanche@itq.upv.es, acorma@itq.upv.es*

Catalyst preparation.

Materials

Magnesium nitrate hexahydrate ($Mg(NO_3)_2·6H_2O$, AR, Sigma-Aldrich); Aluminum nitrate nonahydrate ($Al(NO_3)_3·9H_2O$, AR, Sigma-Aldrich); Sodium carbonate (Na_2CO_3, AR, Sigma-Aldrich); Sodium hydroxide anhydrous pellets (NaOH, AR, Carlo Erba reagent); Sodium nitrate ($NaNO_3$ AR, Sigma-Aldrich).

Commercial zeolites USY (CBV760) and ZSM-5 (CBV5524G) are supplied by Zeolyst International in acid and ammonium form, respectively. The USY zeolite has been used as supplied and the ZSM-5 has been calcined for 3 hours at 500ºC for obtaining the acid form when tested as the pure additive.

Synthesis of Z5-LDH composite

The Z5-LDH composite was synthesized through a modified procedure from previous work.[1, 2] In an initial step, 2.5 g of commercial ZSM-5 (as supplied, in its ammonic form and denoted as Z5-NH_4), with a Si/Al ratio of 25, were dispersed in 200 mL of DI water, and then 2.65 g of Na_2CO_3 were added to create solution A. A second solution B, containing a mixture of $Al(NO_3)_3·9H_2O$ (4.5 g) and $Mg(NO_3)_2·6H_2O$ (6.15 g) in 192 mL of DI water, was gradually added to solution A while vigorously stirring, maintaining the pH at 10 by dropwise addition of a 1M NaOH solution. After one hour of thorough mixing, the obtained solid was separated by filtration and dispersed in 200 mL of DI water for 30 min. The solid was separated again by filtration and washed. Then, it was stirred in acetone overnight following the Aqueous Miscible Organic Solvent Treatment Method (AMOST), aiming to obtain a higher surface area.[3, 4] Finally, the sample was dried in an oven at 100 °C. To be used as an acid catalyst, the as prepared composite (Z5-LDH-as) was converted into its final protonic form by means of an ion-exchange process with 0.1M NH_4NO_3, at 80°C for 2h under vigorous stirring, followed by filtration, washing, and drying. The procedure was repeated

three times. Finally, the obtained sample was calcined at 550 °C for 2 h using a heating rate of 2 °C/min. The final catalyst was designated as Z5-LDH.

Hydrothermal treatment of the Z5-LDH composite

Hydrothermal treatment of the Z5-LDH composite has been performed in a muffle oven, for 5 h at 750ºC, under 100% steam atmosphere. The sample has been named as Z5-LDH-ST.

Synthesis of pure LDH and calcined LDH

The pure LDH can be synthesized following the same procedure described in the synthesis of the Z5-LDH composite but without adding the zeolite. In order to obtain the LDH-derived mixed oxides, the as prepared LDH material (LDH-as) was calcined at 550 °C for 2 h and named as LDH.

Ion-exchange process for Na-Z5 sample

Na^+ exchanged ZSM-5 (Na-Z5) was prepared by suspending 1 g of the commercial ZSM-5, CBV5524G, in its H^+ form, in 10 mL of DI water containing 0.027 g of sodium nitrate and keeping it under vigorous stirring at 80 °C during 2 h. After that, the solid was separated by filtration, washed, dried and calcined at 550 °C for 2 h. This sample was denoted as Na-Z5.

Catalyst characterization techniques

Powder X-Ray Diffraction (PXRD) patterns were recorded on a PANalytical CUBIX diffractometer equipped with a PANalytica X'Celerator detector using Cu Kα as the radiation source, operating at 45 kV, 40 mA and in the 2θ angle range of 2–60°. The morphology of the samples and elemental composition mapping was studied by Field Emission Scanning Electron Microscopy (FESEM) and Energy Dispersive X-ray Spectroscopy (EDS) using a ZEISS ULTRA 55 model (ZEISS OXFORD instruments). After dissolution of the sample in a HNO3/HCl/HF aqueous solution, Inductively Coupled Plasma Mass Spectrometry (ICP) was

used to determine the catalyst composition on a Varian 715-ES ICP-Optical Emission spectrometer. The textural properties of all the synthesized samples were calculated based on the nitrogen adsorption-desorption isotherms obtained in a Micromeritics ASAP 2020 apparatus at 77 K. The total surface (SBET) area was derived from Brunauer–Emmett–Teller (BET) equation. The t-plot method was applied to determine the micropore volume (V_{micro}) and the external surface area (S_{ext}). The acid properties of the samples were evaluated by means of a Nicolet 710 FTIR spectrometer using pyridine as the probe molecule. The pyridine adsorption-desorption experiments were conducted on self-supported wafers of the sample activated at 400°C and 10^{-2} Pa for 2 h. After activation, the base spectrum was recorded, and pyridine vapor was introduced in the vacuum IR cell and adsorbed onto the sample. Then, pyridine was desorbed under vacuum over three consecutive one-hour periods of heating at 150, 250 and 350°C, each of them followed by the IR measurement at room temperature. In addition, the spectra were normalized by the area of the corresponding Si–O overtones in the region of 1755–2100 cm^{-1}. NH_3-TPD experiments were carried out using Micromeritics 2900 apparatus. A calcined sample (100 mg) was activated by heating to 400 °C for 2 h in an oxygen flow and for 2 h in an argon flow. Subsequently, the samples were cooled down to 176 °C, and NH_3 was adsorbed. NH_3 desorption was monitored with a quadrupole mass spectrometer (Balzers, Thermo Star GSD 300T) while the temperature of the sample was ramped at 10 °C min^{-1} in a helium flow. Total ammonia adsorption was measured by repeated injection of calibrated amounts of ammonia at 100 °C until saturation. The mass spectrometer was set at m/e = 15 for ammonia detection to prevent the interference of water fragmentation.

Catalytic performance

The catalytic cracking experiments were performed in a microactivity test (MAT) unit, at 520 °C and 30 s time on stream (TOS). The reaction zone and product recovery system of the MAT unit have been designed according to ASTM D-3907. More details on the reaction system can be found in previous a work [5]. A

commercial USY zeolite (CBV760, Zeolyst Int.), denoted as FAU, was used as the main catalyst (0.3 g). When used as additives for the catalytic cracking of the heavy oil fractions, Z5 and Z5-LDH were added to the main FAU catalyst in a 20 wt.% as separated particles mixed in a single bed. In all cases, the catalyst bed was completed with SiO_2 (Silica gel 60, 0.2-0.5 mm, Sharlau) to a total amount of 3 g and the conversion was varied by changing the catalyst-to-oil (C/O) ratio between 0.15 to 0.67, referred to the amount of FAU, by keeping constant the amount of catalyst and adjusting the feedstock amount of oil. For studying the intrinsic contribution of the ZSM-5 and LDH-derived mixed oxides to VGO cracking, in absence of the FAU base catalysts, four different cases were compared, the calcined ZSM-5 zeolite alone, Z5 (0.3 g), the final acid Z5-LDH composite (0.48 g), a physical mixture of Z5 and calcined LDH, Phy (0.3 and 0.18 g, respectively), and a double-bed configuration (2-Bed) with 0.18 g of the calcined LDH in the first bed (top) and 0.3 g of Z5 in the second one (bottom). In all four cases, the catalyst bed was completed with SiO2 (Silica gel 60, 0.2-0.5 mm, Sharlau) to a total amount of 3 g, and the C/O ratios, calculated based on the amount of Z5, were varied between 0.15 and 0.67. The gas products were analyzed using a Shimadzu GC-2014 equipped with two detectors including a thermal conductivity detector (TCD) and a flame ionization detector (FID) for analysis of permanent gases (H_2 and N_2), and C_1–C_6 hydrocarbons, respectively. The accumulated liquid products were analyzed by simulated distillation in a Shimadzu Nexis GC-2030.

In this work, two different feedstocks were used, a vacuum gas oil (VGO) and an atmospheric residue (ATM-residue). The properties of the feeds are enclosed in Table S1.

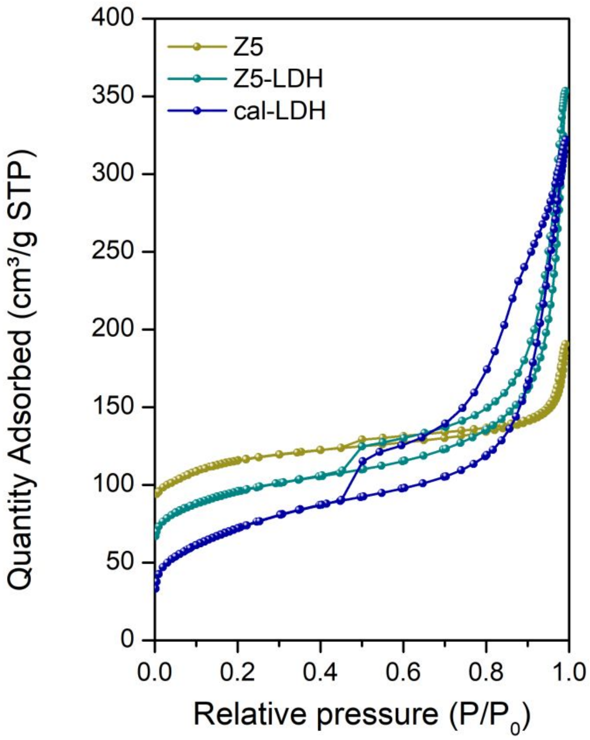

Fig. S1 N_2 adsorption-desorption isotherms of Z5 zeolite, Z5-LDH composite and cal-LDH. The same scale is applied to the three measurements, with no offset among the different isotherms.

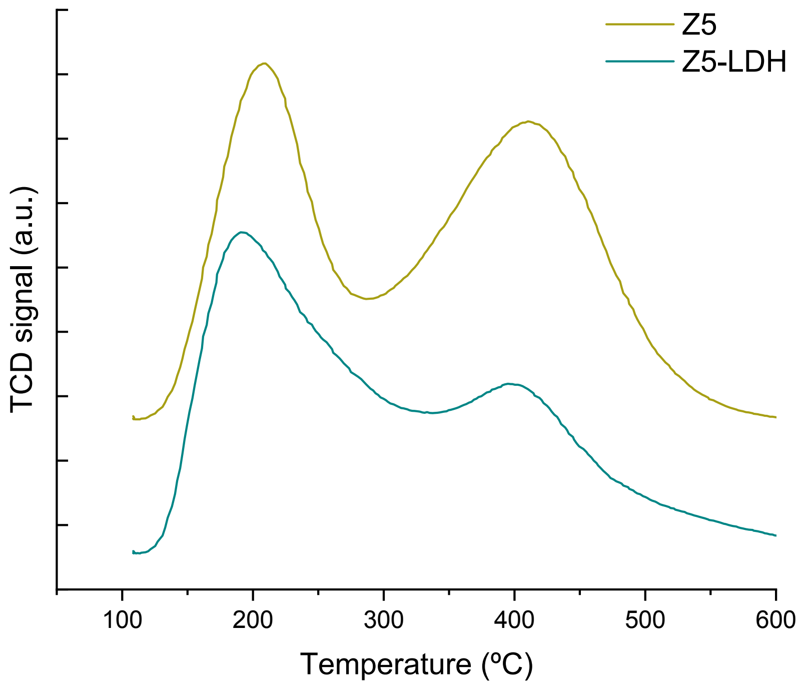

Fig. S2 NH_3-TPD profiles obtained for Z5 zeolite and Z5-LDH composite.

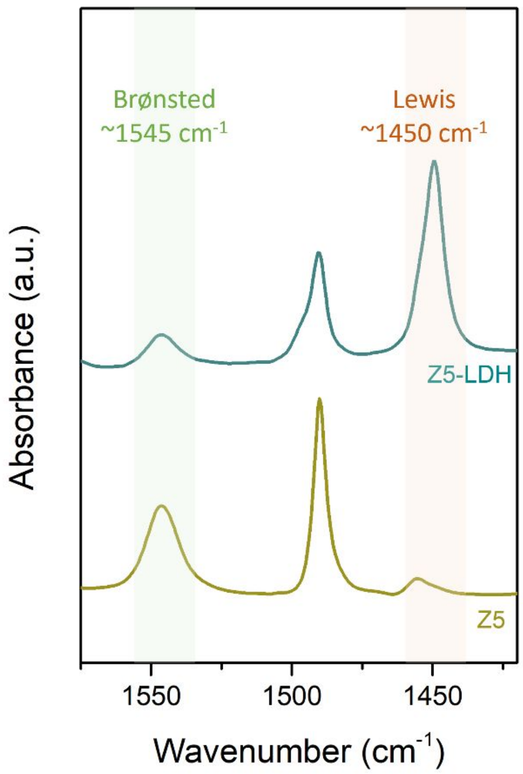

Fig. S3 FT-IR spectra of pyridine adsorbed on Z5 and Z5-LDH samples after desorption at 150 °C.

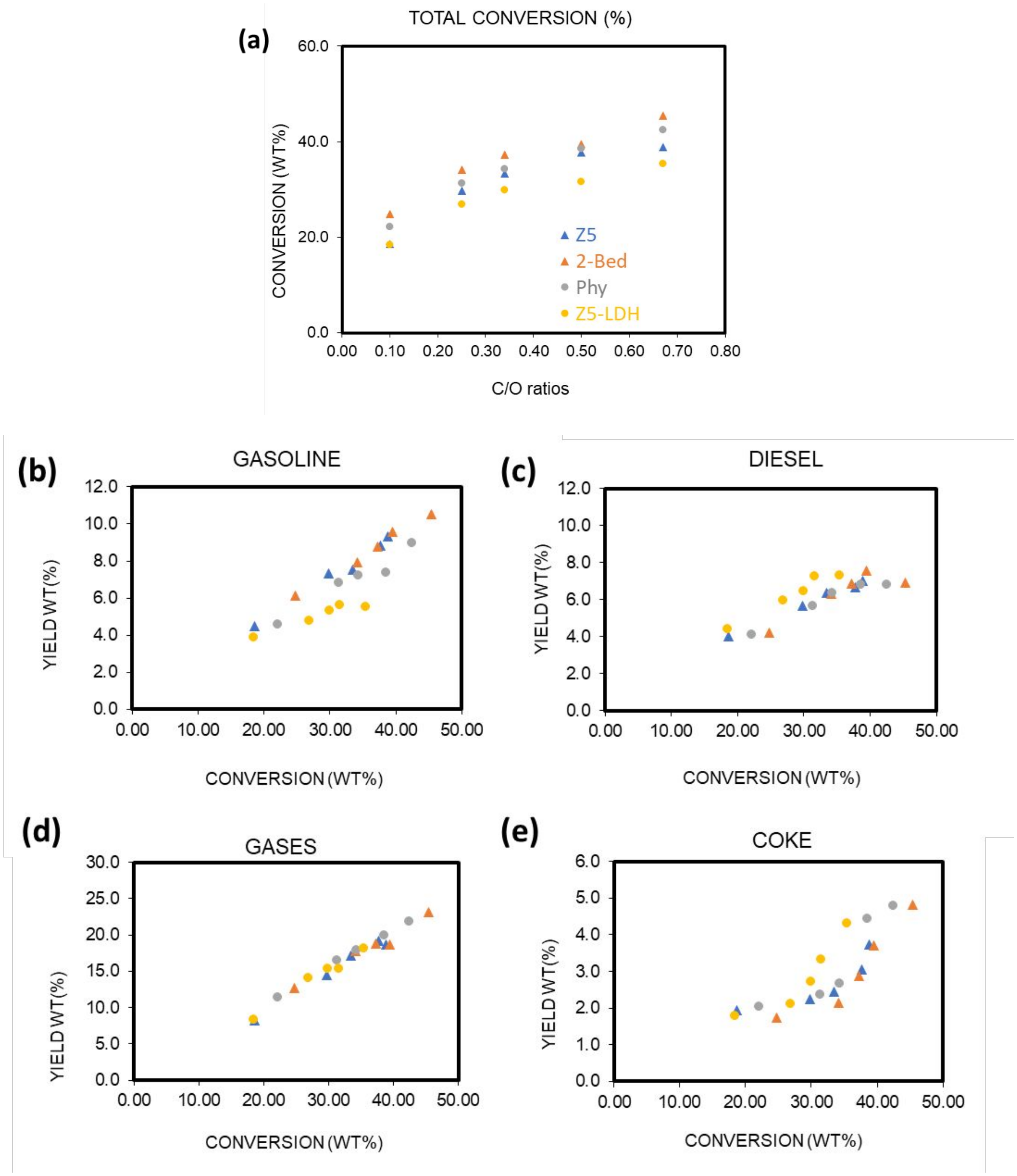

Fig. S4 VGO conversion (a) and product distribution (b-e) obtained with Z5, Z5-LDH, a physical mixture (Phy), and a double-bed configuration (2-Bed) of Z5 and cal-LDH in VGO cracking reaction at 520 °C and 30 s of TOS.

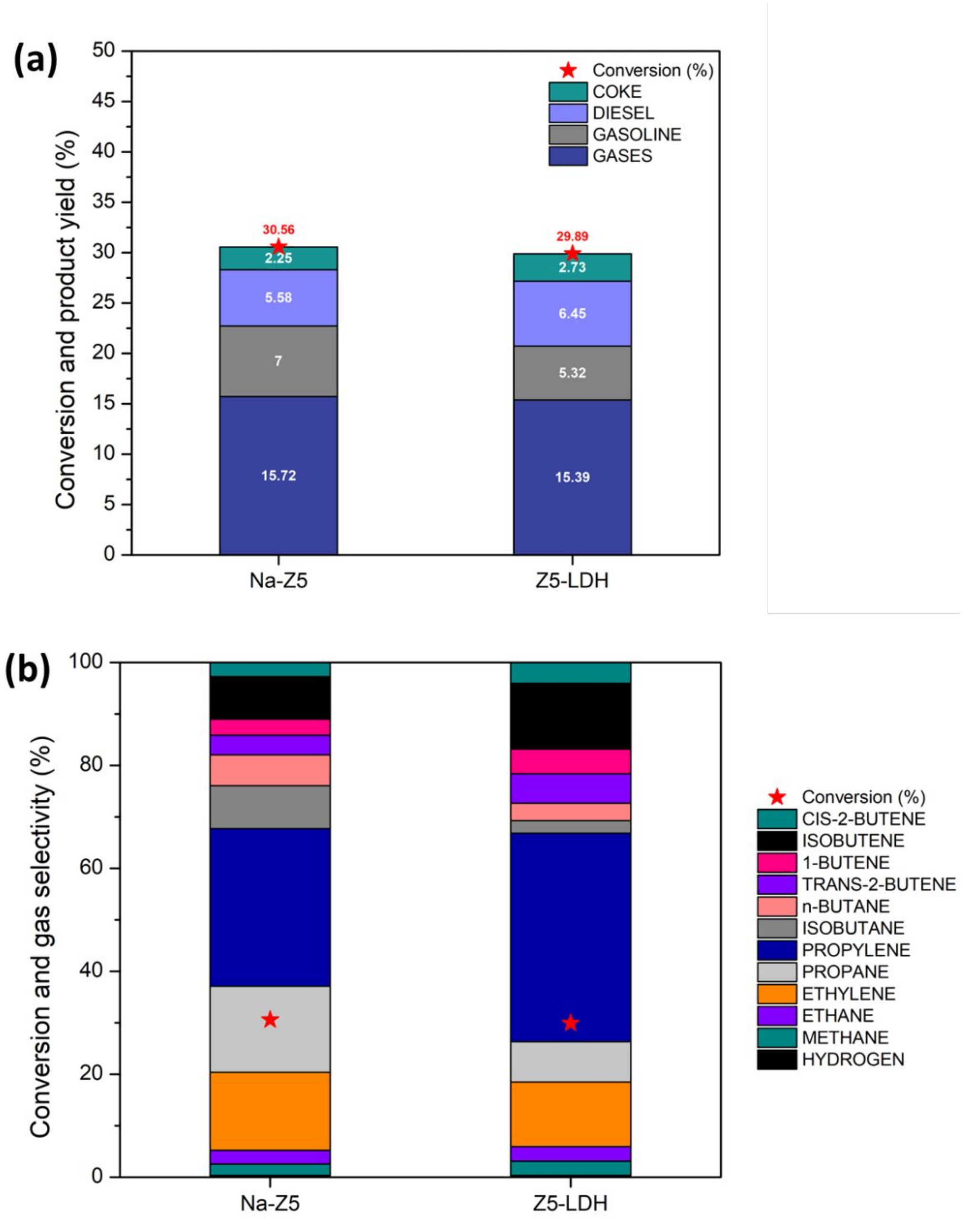

Fig. S5 VGO conversion and overall product distribution (a), and product distribution within the gas fraction (b) obtained with Na-exchanged ZSM-5 (Na-Z5) and Z5-LDH composite in VGO cracking reaction at C/O of 0.34, T=520 °C and 30 s of TOS.

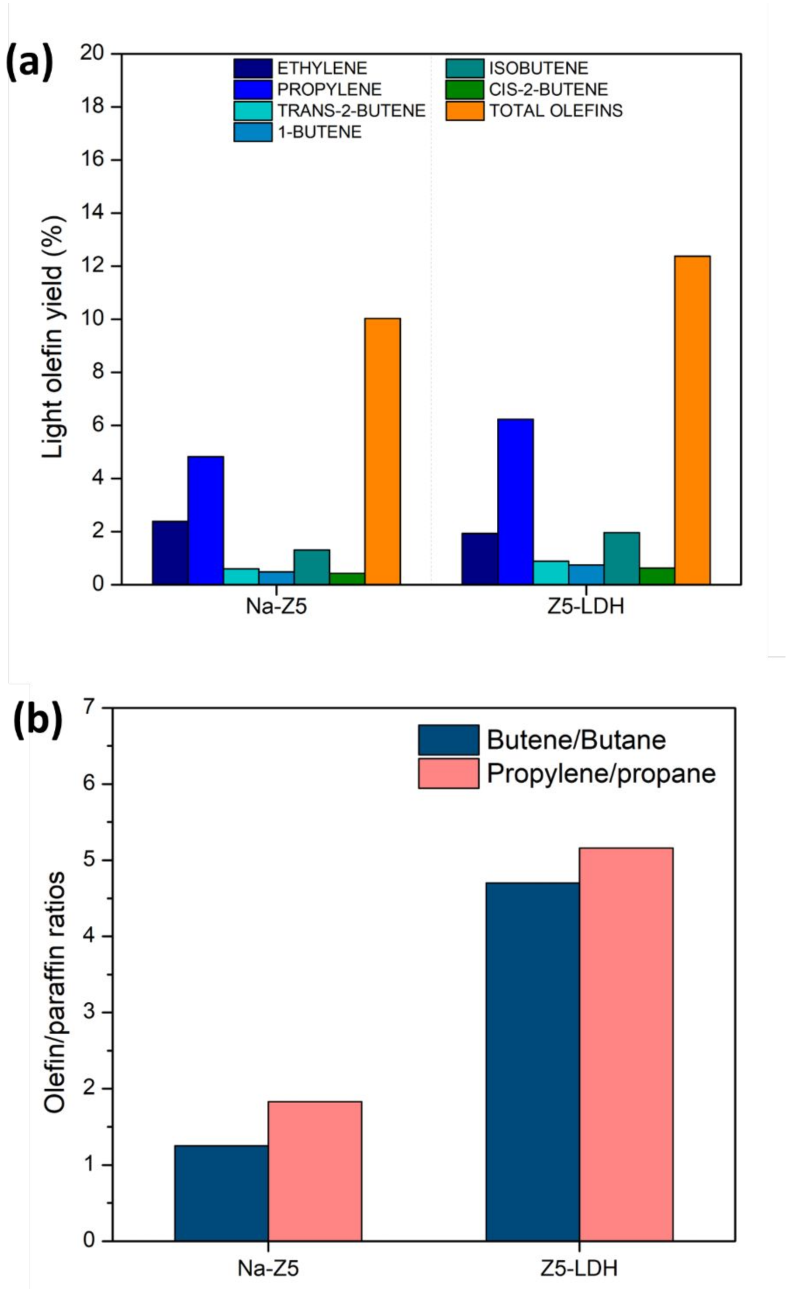

Fig. S6 Light olefin yields (a) and propylene/propane and butene/butane ratios (b) obtained with Na-exchanged ZSM-5 (Na-Z5) and Z5-LDH composite in VGO cracking reaction at C/O of 0.34, T=520 °C and 30 s of TOS.

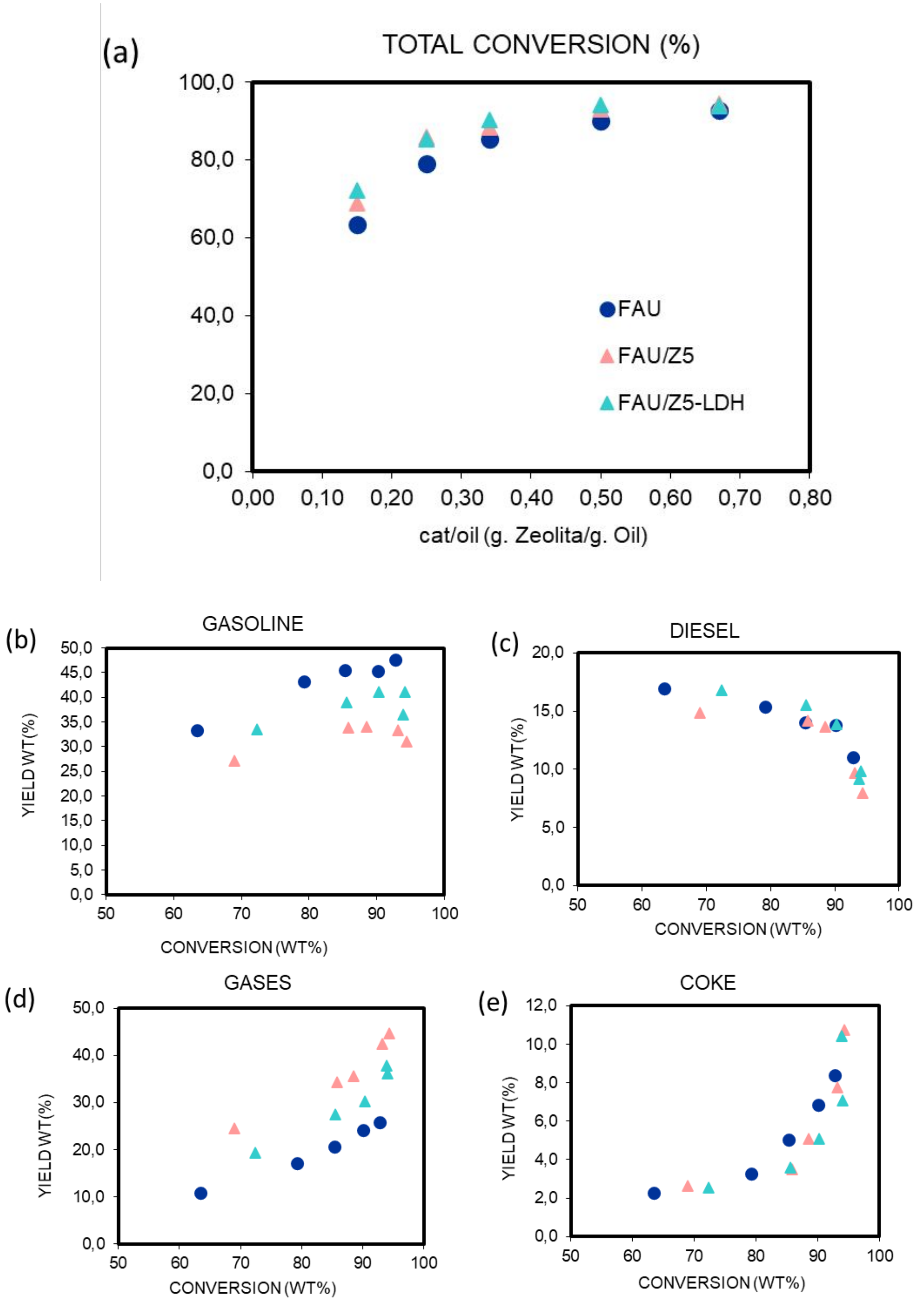

Fig. S7 VGO conversion (a) and product distribution (b-e) of catalysts with different additives in VGO cracking reaction at 520 °C and 30 s of TOS.

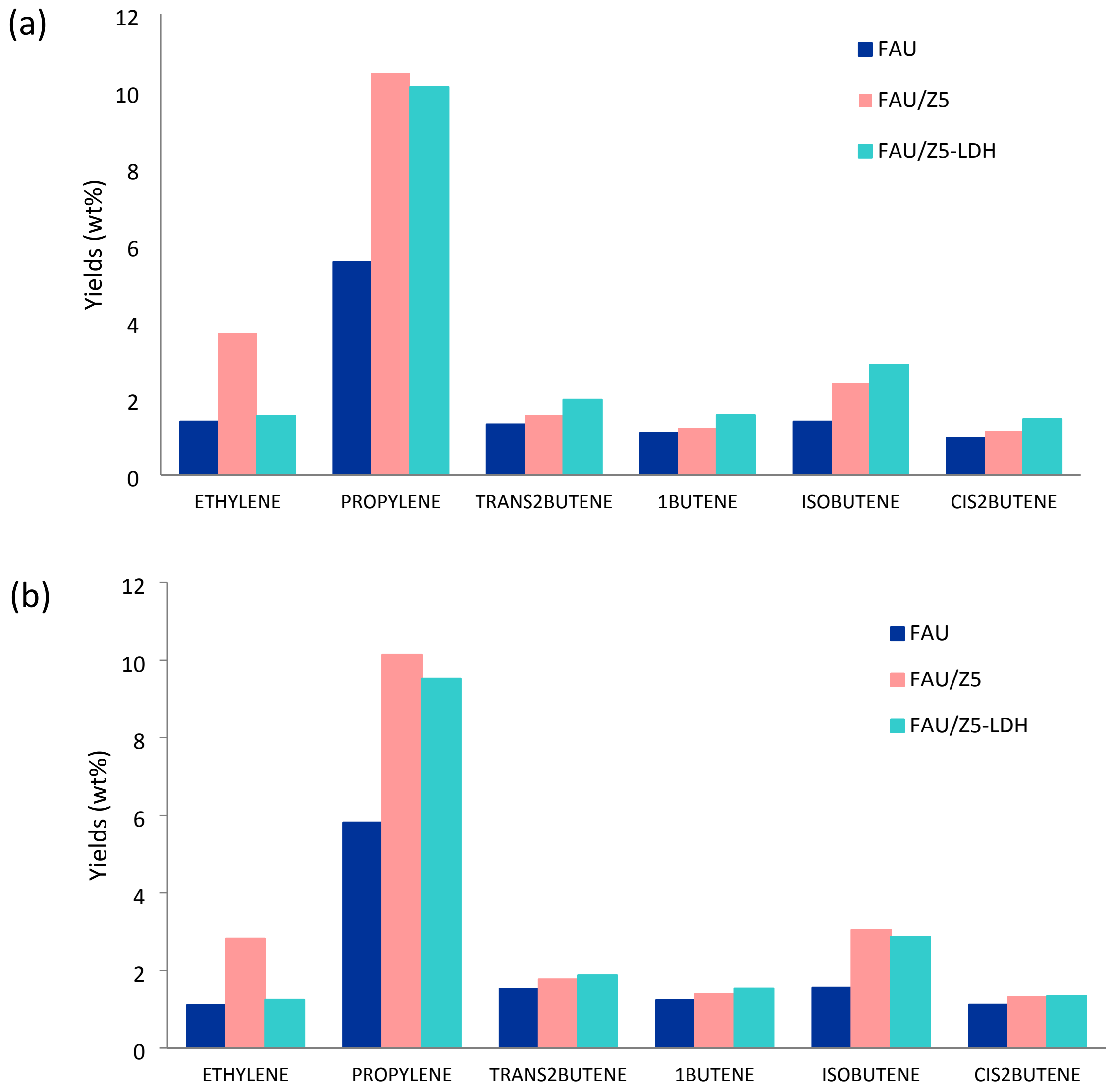

Figure S8. Comparison of light olefin yields between different catalysts at a total conversion of ~90 % in the cracking reaction of (a) VGO and (b) atmospheric residue. T=520 °C, TOS=30 s.

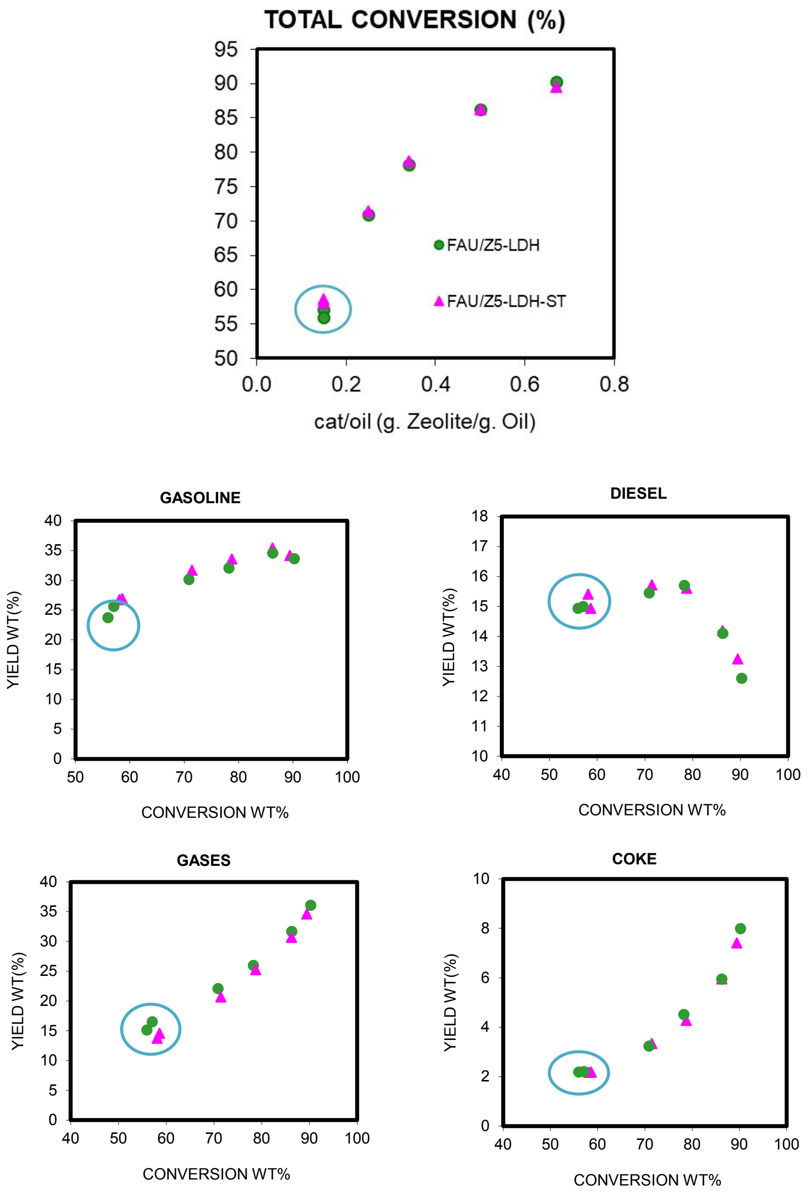

Figure S9. VGO conversion (a) and product distribution (b-e) of catalysts with additives Z5-LDH (calcined) and Z5-LDH-ST (steamed) in VGO cracking reaction at 520 °C and 30 s of TOS.

Table S1. Characteristics of the vacuum gas oil and the atmospheric residue.

	VGO	ATM-residue
DENSITY (15ºC, g/cc)	0.9012	0.9289
ANILINE POINT (ºC)	94	87
ASPHALTHENES (wt%)	0.05	1.1
SULFUR (%wt)	0.86	0.61
NITROGEN (ppm)	938	692
VISCOSITY (100ºC, cst)	7	17.4
Ni (ppm)	0.2	3
V (ppm)	0.2	5
ASTM 1160 (ºC)		
5%	382	330
10%	396	364
30%	419	430
50%	434	481
70%	459	542
90%	512	642
HYDROGEN (%)	12.565	12.268
K (UOP)	12.082	11.917
AROMATIC C (ndM)%	17.1	17.3
NAPHTHENIC C(ndM)%	19.1	22.5
PARAFFINIC CARB(ndM)%	63.8	60.5

Table S2. Chemical composition of synthesized Z5-LDH sample as determined by ICP.

Sample	Element composition				LDH content (%wt) [a]
	Si (%wt)	Al (%wt)	Mg (%wt)	Na (%wt)	
Z5	96.3	3.7	-	-	-
Z5-LDH	59.6	16.2	24.2	-	38.1

[a] Data was calculated from ICP results.

Table S3 Relative crystallinity (%) of Z5-LDH with respect to pure commercial ZSM-5 (Z5)

Sample	Relative crystallinity (%) [a]
Z5	100
Z5-LDH	61.4

[a] Data was obtained from XRD measurement. Relative crystallinity was calculated by integrating the peak area in the range of 22.5˚ - 25˚divided by the corresponding peak area of the pristine Z5.

Table S4 Textural properties of the USY catalysts and the different additives.

Catalyst	S_{BET}[a] (m^2/g)	S_{micro}[b] (m^2/g)	S_{ext}[c] (m^2/g)	V_{total}[d] (cm^3/g)	V_{micro}[e] (cm^3/g)	V_{meso}[f] (cm^3/g)
FAU	768	690	78	0.54	0.34	0.20
Z5	380	338	42	0.29	0.17	0.07
Z5-LDH	321	232	89	0.53	0.11	0.15
Calcined LDH	258	163	95	0.48	0.08	0.24

[a] S_{BET} (BET specific surface area); [b] S_{micro} (micropore surface area); [c] S_{ext} (external surface area) obtained by *t*-plot method; [d] V_{total}(total pore volume); [e] V_{micro} (micropore volume) obtained by *t*-plot method; [f] V_{meso} (mesopore volume) = BJH Adsorption cumulative volume of pores between 17 and 300 Å

Table S5 Acidity of the Z5-LDH and Na-Z5 as determined by FT-IR combined with pyridine adsorption-desorption.

Sample	Lewis acid sites [a] (μmol/g of catalyst)	Brønsted acid sites [b] (μmol/g of zeolite)
	T= 150 °C	T= 150 °C
Na-Z5	29	304
Z5-LDH	506	290

[a] Lewis acid sites were calculated based on the total weight of catalyst.
[a] Brønsted acid sites were calculated based on the weight of zeolite.

References

[1] C. Rodaum, A. Thivasasith, D. Suttipat, T. Witoon, S. Pengpanich, and C. Wattanakit, "Modified acid-base zsm-5 derived from core-shell ZSM-5@ aqueous miscible organic-layered double hydroxides for catalytic cracking of n-pentane to light olefins," ChemCatChem, vol. 12, no. 17, pp. 4288-4296, 2020, doi: 10.1002/cctc.202000860.

[2] C. Chen, C. F. H. Byles, J.-C. Buffet, N. H. Rees, Y. Wu, and D. O'Hare, "Core–shell zeolite@aqueous miscible organic-layered double hydroxides," Chem. Sci., vol. 7, no. 2, pp. 1457-1461, 2016, doi: 10.1039/C5SC03208C

[3] H. Suo, C. Chen, J.-C. Buffet, and D. O'Hare, "Dendritic silica@aqueous miscible organic-layered double hydroxide hybrids," Dalton trans., vol. 47, no. 46, pp. 16413-16417, 2018, doi: 10.1039/C8DT04128H.

[4] Q. Wang and D. O'Hare, "Large-scale synthesis of highly dispersed layered double hydroxide powders containing delaminated single layer nanosheets," Chem. Commun., vol. 49, no. 56, pp. 6301-6303, 2013, doi: 10.1039/C3CC42918K.

[5] A. Corma, C. Martínez, F. Melo, L. Sauvanaud, and J. Carriat, "A new continuous laboratory reactor for the study of catalytic cracking," Appl. Catal., A, vol. 232, no. 1-2, pp. 247-263, 2002, doi: 10.1016/S0926-860X(02)00110-2.
